# Application of Metagenomic Next-Generation Sequencing in *Mycobacterium tuberculosis* Infection

**DOI:** 10.3389/fmed.2022.802719

**Published:** 2022-04-01

**Authors:** Yaoguang Li, Mengfan Jiao, Ying Liu, Zhigang Ren, Ang Li

**Affiliations:** ^1^Department of Infectious Diseases, The First Affiliated Hospital of Zhengzhou University, Zhengzhou, China; ^2^Gene Hospital of Henan Province, Precision Medicine Center, The First Affiliated Hospital of Zhengzhou University, Zhengzhou, China

**Keywords:** metagenomic next-generation sequencing, tuberculosis, *Mycobacterium tuberculosis*, precise treatment, infectious disease

## Abstract

The fight against *Mycobacterium tuberculosis* (MTB) has been going on for thousands of years, while it still poses a threat to human health. In addition to routine detections, metagenomic next-generation sequencing (mNGS) has begun to show presence as a comprehensive and hypothesis-free test. It can not only detect MTB without isolating specific pathogens but also suggest the co-infection pathogens or underlying tumor simultaneously, which is of benefit to assist in comprehensive clinical diagnosis. It also shows the potential to detect multiple drug resistance sites for precise treatment. However, considering the cost performance compared with conventional assays (especially Xpert MTB/RIF), mNGS seems to be overqualified for patients with mild and typical symptoms. Technology optimization of sequencing and analyzing should be conducted to improve the positive rate and broaden the applicable fields.

## Introduction

Tuberculosis (TB), an infectious disease that has plagued humankind for thousands of years, disturbingly causes an estimated 10 million people to fall ill in the world in 2019 (1). Among the 10 million cases, the proportion of drug resistance against either rifampicin (RIF) or isoniazid (INH) is approximately 15% (2). It still exists as a dilemma to detect *Mycobacterium tuberculosis* (MTB) promptly and determine the drug resistance definitely to guide clinical treatment (3), which is considered one of the leading causes of the high mortality rate (4). Therefore, the World Health Organization is calling for taking early diagnosis and comprehensive drug susceptibility testing (DST) as a priority and key component for TB care (2, 5). To obtain a rapid and accurate diagnosis of the culprit microorganism, scientists and clinicians spare no effort to modify routine detections or apply advanced technologies to maximize detection efficiency.

Metagenomic next-generation sequencing (mNGS) is increasingly reckoned as a comprehensive and hypothesis-free test, which outperformed in pathogen detection (6). Without isolating specific pathogens, it directly extracts all the hereditary material fragments (DNA or RNA) from clinical samples and sequences them simultaneously and independently. After that, experimenters compare the detected sequences with the reliable database that comprehensively covers known pathogenic microorganisms (7). Besides detecting MTB, it shows potential to find co-infection pathogens, suggest underlying tumors, or determine drug resistance in one run, which is significant for overall diagnosis and timely treatment.

Herein, we will briefly summarize the advantages and deficiencies of routine detections, introduce the recent progress of mNGS in TB, concentrate on practical considerations and corresponding solutions, and finally, look forward to broader application in the future.

## *Mycobacterium tuberculosis* Infection and Routine Detections

Infected by MTB does not imply the inevitable onset of typical symptoms (8). Clinical manifestations vary from person to person according to the strain virulence and host immunocompetence, presenting as active TB and latent TB infection (LTBI) (9). Active TB can harm multiple organs, including the lung, brain, joint, and bone, even resulting in disseminated damage. LTBI accounts for 90% of human infections, potentially affecting 2 billion individuals (1). It poses a potential hazard to public health security (10). When the infected individuals are in a low immune state [such as co-infected with HIV (11) or coronavirus disease 2019 (COVID-19) (12)], the newly activated TB is more aggressive and awkward to cope with. Additionally, the emergence and transmission of drug-resistant strains have challenged the prevention and treatment, characterized as rifampin resistance (RR), multidrug resistance (MDR), or extensive drug resistance (XDR). They greatly increase the medical expense and financial burden (13).

Given the characteristics of MTB [thick-walled, weakly gram-positive, and acid-fast and long growth cycle (14)] and complex host immunoreaction, routine detections show limited effects (Table 1). Tests universally used for MTB are culture and acid-fast staining (AFS), providing intuitionistic evidence (15). While limited by a long growth circle and low positive rate, it is not so dependent on the clinic (16). Imaging examination, such as chest radiography or computed tomography (CT), plays a vital role in diagnosis and follow-up (17) but is not specific in extrapulmonary TB (18). In recent decades, molecular diagnosis has made significant breakthroughs. Polymerase chain reaction (PCR) after extracting nucleic acid directly from clinical samples is widely acknowledged (1). Xpert MTB/RIF (Xpert) has been applied to detect MTB and RIF resistance (19). Also, the improved version, Xpert MTB/RIF Ultra (Ultra), shows better performance with higher sensitivity and increases the detection rate of immunodeficient patients with HIV infection (20). Researchers are also pushing the boundaries to apply advanced technologies to detect MTB, such as CRISPR-based diagnostic tests for MTB (CRISPR-MTB) (21). Additionally, indicating cellular immune response, tuberculin skin test (TST) and interferon-γ release assay (IGRA) can be used to identify LTBI (22). However, they have low sensitivity in immunocompromised populations, and they show poor discrimination between LTBI and active TB (22, 23) as well as TB and Bacille Calmette-Guerin (BCG) vaccination (24).

**TABLE 1:** Summary of advantages and limitations of routine detections for *Mycobacterium tuberculosis* (MTB).

Detections	Advantages	Limitations
Culture	• Gold standard; • Low cost; • Drug susceptibility testing.	• Long growth circle (it takes up to 8 weeks to grow into visible colonies on solid culture media) (16); • Low sensitivity and poor positive rate.
Acid-fast staining	• Low cost; • Short turnaround time (the average time is 16.6 h) (90).	• Hard to distinguish between *Mycobacterium leprae* and non-tuberculous *Mycobacteria* (NTM) (96); • Tend to receive negative results in HIV-infected patients or children, who bear low bacterial load (97); • Low sensitivity and poor positive rate.
Imaging examination	• Assist in diagnosis and follow-up (17, 18)	• Atypical when co-infection or low immune status occur (17); • Not specific in extrapulmonary TB (18).
Xpert MTB/RIF	• Short turnaround time (the average turnaround time is 24.1 h) (90); • Drug resistance detection (against RIF) (98); • High sensitivity and specificity (20)	• Only specific sites can be detected (20, 99); • Xpert has limited sensitivity in HIV patients with miliary lung infiltrates, mainly due to paucibacillary specimens (20, 99); • Better testing might not improve the outcomes (100, 101).
TST and IGRA	• Low cost; • Identify LTBI (22).	• Low sensitivity in immunocompromised populations; • False-positive in patients vaccinated with Bacillus Calmette-Guerin (BCG) via TST (24); • Unable to differentiate between LTBI and active TB (22, 23)

Drug resistance detection is necessary for the confirmation of RR/MDR/XDR-TB strain, generally using molecular tools (1). Xpert, an integrated hands-free real-time PCR testing to amplify the RR-determining region (RRDR) of the MTB rpoB gene, provided us with a new perspective of detecting certain sites (25). It simultaneously detects MTB and RR using the molecular technique, which revolutionarily shortened the detection time to 2 h (26, 27). The improved version, Ultra, incorporates two different multicopy amplification targets, namely, IS6110 and IS1081, and RRDR of the rpoB gene (28). However, routine drug resistance detections are only designed to detect partial known mutation sites.

## Metagenomic Next-Generation Sequencing Workflow for *Mycobacterium tuberculosis* Infection

With the booming development of sequencing technology, mNGS has begun to show presence as a comprehensive and powerful detection, which detects nucleic acid fragments with high sensitivity and specificity (29, 30). The most striking feature is that it can sequence all the nucleic acid components directly from the clinical samples (31), allowing for a hypothesis-free detection and comprehensive diagnosis.

The dominant sequencing technology in the current market is the next-generation short-read and long-read sequencing approach (32). Also, the most representative platforms are the Illumina sequencers (Illumina MiSeq and iSeq100) and the Nanopore sequencers (Oxford Nanopore MinION), respectively (33). Short-read platforms are widely used in the clinic for satisfying stability; while the long-read platform has been widely adopted in development prospects for detecting drug resistance, surveilling epidemic outbreaks, and so on (34–36).

**FIGURE 1:**
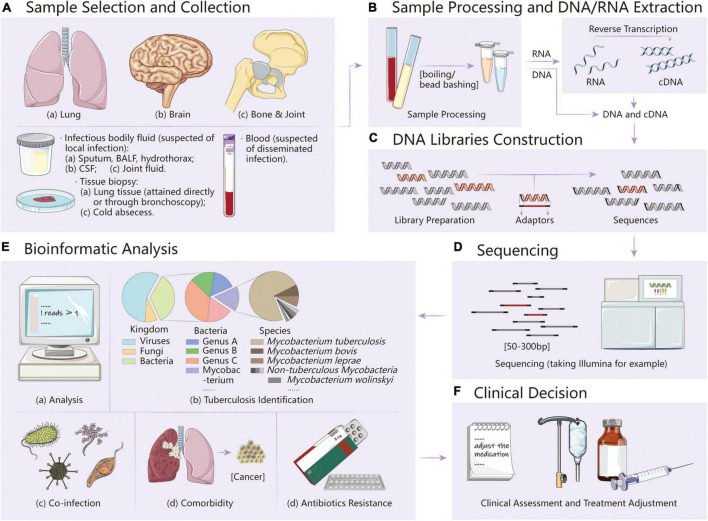
**(A)** Sample selection and collection. Infectious fluids and tissues can be taken under the evaluation of clinicians. **(B–D)** Experimental procedures (wet lab) of mNGS, mainly including sample processing and DNA/RNA extraction, DNA libraries construction, and sequencing. **(E)** Bioinformatic analysis (dry lab) of mNGS. With the analysis pipeline modification, it is promising to determine tuberculosis, co-infection, comorbidity, and antibiotics resistance simultaneously. **(F)** Clinical decision. After obtaining the report, clinical assessment and treatment adjustment can be made promptly.

The process of mNGS mainly includes two parts: experimental procedures (wet lab) and bioinformatic analysis (dry lab). After collecting suitable samples under the guide of clinicians, the wet lab workflow can be roughly divided into three stages, namely, DNA/RNA extraction, library preparation, and sequencing (33) (Figure 1). Also, the dry lab is the last step to generate the final report. Brief bioinformatic procedures mainly start from raw input FASTQ files, followed by quality and low-complexity filtering, human host subtraction, and microorganism identification aligning to reference databases (7). Raw data generated from different platforms should be, respectively, separated and strictly quality-controlled (Q30 qualified) (33). To obtain clean reads, low-quality reads can be removed by the Trimmomatic or fastp tools, low-complexity regions can be masked by DustMasker, and duplicated reads can be removed by PRINSEQ (37, 38). Then, it is necessary to remove human host reads to shorten analysis time with mapping software (Bowtie 2, BWA, and HISAT2) or software specialized in removing host sequences (BMTagger and CS-SCORE) (39, 40). Taxonomic classification is based on the sequence similarity with the alignments with reference genomes. Only the alignments that fulfill the abovementioned criteria were used for further pathogen identification. Finally, the data must be filtered by a certain threshold. For MTB, choosing genus-specific read numbers ≥ 1 as the reporting threshold can result in credible reports (14).

With the technical advance, the turnaround time has been dramatically shortened, making it possible to get a report within 24 h (no more than 23 h on Illumina and less than 5–7 h on Nanopore) (41).

In addition to comprehensive testing, targeted next-generation sequencing (NGS) is playing an alternative role (5). Targeted NGS is focused on specific genomic regions in a genome, selecting gene regions of suspected pathogen or specific phenotype (5). Therefore, it is of great importance for the low-load pathogen, from which MTB detection can benefit a lot. Also, it offers a new thread to drug resistance detection, especially on the Nanopore sequencers (42–44).

## Diagnostic Metagenomic Next-Generation Sequencing for *Mycobacterium tuberculosis* Infection

### *Mycobacterium tuberculosis* Detection and Identification

Regarding different clinical manifestations, disease stages, and lesion organs, relevant sample types are available for testing, including various infectious body fluids and tissues (Table 2).

**TABLE 2:** Effectiveness of metagenomic next-generation sequencing (mNGS) for MTB detection.

References	Research type and sample size	Research conclusions and results
Li et al. (46)	• Type: A * • Sample: lung tissue (*n* = 20) • Pre-ATT samples (not mentioned) • MTBC mNGS positive (*n* = 4) • Pathogenic TB diagnoses (*n* = 4) Diagnosis basis: AFS, Xpert, PCR, etc.	• MNGS showed the highest Spe and PPV for MTBC when compared with histopathology method. • For MTBC lung tissue mNGS: compared with smear: Sen: 100.0% (19.8–100.0%), Spe: 88.9% (63.9–98.1%); compared with histopathology: Sen: 100.0% (31.0–100.0%), Spe: 94.1% (69.2–99.7%).
Miao et al. (6)	• Type: C • Sample: all samples (*n* = 511) • Pre-ATT samples (not mentioned) • TB mNGS positive (*n* = 42) • Total TB diagnoses (*n* = 92) Pathogenic TB diagnoses (*n* = 25) Diagnosis basis: culture, clinical criteria	• MNGS outperformed culture, especially for MTB [odds ratio = 4 (1.7–10.8)]. • For MTB mNGS: Sen: 45.7% (42/92). • For NTM mNGS: Sen: 29.8% (17/57).
Wang et al. (51)	• Type: A + B • Sample: CSF (*n* = 29)^$^ • Pre-ATT samples (not mentioned) • TB mNGS positive (*n* = 42) • Total TB diagnoses (*n* = 23) Pathogenic TB diagnoses (*n* = 12) Clinical TB diagnoses (*n* = 11) Diagnosis basis: culture, AFS, PCR, clinical criteria	• Combining mNGS and conventional methods (culture, AFS, PCR) increased the detection rate to 95.65%. • For MTBC CSF mNGS: Sen: 66.67% (8/12); Spe: 100% (6/6); PPV: 100% (8/8); NPV: 60% (6/10); accuracy: 77.78% (14/18).
Zhou et al. (62)	• Type: B • Sample: all samples (*n* = 105) Pulmonary samples (*n* = 27) CSF samples (*n* = 49) Other extrapulmonary samples (n = 29) • Pre-ATT samples (*n* = 27) • TB mNGS positive (*n* = 20) • Total TB diagnoses (*n* = 45) Diagnosis basis: culture, Xpert, PCR, clinical criteria	• Combining mNGS and Xpert improved the etiology diagnosis, increased specificity from 44% (20/45, 30–60%) to 60% (27/45, 44–74%); • Empirical ATT reduced diagnostic efficacy of culture, Xpert, and mNGS. • For MTB mNGS: Sen: 44% (20/45, 30–60%), Spe: 98% (59/60, 91–100%); • For MTB pulmonary samples mNGS: Sen: 62% (8/13, 32–86%); Spe: 100% (14/14, 77–100%); • For MTB CSF mNGS: Sen: 44% (7/16, 20–70%); Spe: 97% (32/33, 84-99%); • For MTB other extrapulmonary samples mNGS: Sen: 31% (5/16, 11–59%); Spe: 100% (13/13, 75–100%).
Xing et al. (77)	• Type: A • Sample: CSF (*n* = 213) Pre-ATT samples (not mentioned) • TB mNGS positive (shown rightward) • Total TB diagnoses (*n* = 44) Pathogenic TB diagnoses (*n* = 6) Clinical TB diagnoses (*n* = 38) Diagnosis basis: AFS, Xpert, clinical criteria	• When the genus-specific read number ≥ 1 was considered MTB positive, the AUC (61.9%, 51.6–72.1%) was largest. • Given high specificity (96.4%, 163/169) of mNGS in the diagnosis of TBM, it allows a negative mNGS test to be used as one of the diagnostic methods to exclude TBM. • For MTB CSF mNGS: if genus-specific read numbers ≥ 1, 2, 3, 5, 10 was considered positive; the positive consistency rates were 27.3, 20.5, 18.2, 13.6, 6.8%; the negative consistency rates were 96.4, 97.6, 98.2, 99.4, and 100%; the total consistency rates were 82.2, 81.7, 81.7, 81.2, and 80.8%, respectively.
Yan et al. (50)	• Type: A + B • Sample: CSF (*n* = 51) • Pre-ATT sample (*n* = 51) • TB mNGS positive (*n* = 38) • Total TB diagnoses (*n* = 45) Pathogenic TB diagnoses (*n* = 38) Clinical TB diagnoses (*n* = 7) Diagnosis basis: culture, AFS, PCR, Xpert, clinical criteria (102)	• Patients with a significant increase in CSF cell number and protein quantification might have a higher likelihood of positive MTB detection of mNGS. • For MTB CSF mNGS: Sen: 84.44% (38/45, 69.94–93.01%); Spe: 100% (6/6, 51.68–100%); PPV: 100.0% (40/40, 88.57–100%); NPV: 46.15% (6/13, 20.40–73.88%).
Chen et al. (70)	• Type: B. • Sample: all samples (*n* = 70) Pulmonary samples (*n* = 37) Extrapulmonary samples (*n* = 33) • Pre-ATT samples (not mentioned) • TB mNGS positive (*n* = 25) • Total TB diagnoses (*n* = 36) Pathogenic TB diagnoses (*n* = 36) Diagnosis basis: pathological test, PCR	• Combining mNGS and culture or Xpert improved Sen to72.2% (26/36, 54.6–85.2%), higher than only mNGS (66.7%, 24/36, 48.9–80.9%), showing the potential for clinical application in TB. • For MTB pulmonary samples mNGS: Sen: 82.4% (14/17, 55.8–95.3%); Spe: 100% (20/20, 80.0–100.0%); PPV: 100% (14/14, 73.2–100.0%); NPV: 87.0% (20/23, 65.3–96.6%); Youden index: 82.4%; • For MTB extrapulmonary samples mNGS: Sen: 47.4% (9/19, 25.2–70.5%); Spe: 92.9% (13/14, 64.2–99.6%); PPV: 90.0% (9/10, 54.1–99.5%); NPV: 56.5% (13/23, 34.9–76.1%); Youden index: 40.3%.
Jin et al. (30)	• Type: B. • Sample: all samples (*n* = 820) Pulmonary samples (*n* = 477) Extrapulmonary samples (*n* = 343) Pre-ATT samples (not mentioned) • TB mNGS positive (*n* = 76) • Total TB diagnoses (*n* = 125) Pathogenic TB diagnoses (*n* = 64) Clinical TB diagnoses (*n* = 61) Diagnosis basis: culture, Xpert, PCR, clinical criteria	• mNGS may be a promising technology for sputum-negative PTB and tuberculous serous effusion. • For MTB mNGS: Sen: 49.6% (62/125, 40.6–58.6%), Spe: 98.3% (683/695, 96.9–99.1%); • For MTB pulmonary samples mNGS: Sen: 58.5% (31/53, 44.2–71.6%); Spe: 98.3% (417/424, 96.5–99.3%); • For MTB extrapulmonary samples mNGS: Sen: 43.1% (31/72, 31.6–55.2%), Spe: 98.2% (266/271, 95.5–99.3%).
Shi et al. (61)	• Type: B • Sample: BALF (*n* = 110) • Pre-ATT samples (not mentioned) • TB mNGS positive (*n* = 24) • Total TB diagnoses (*n* = 48) Pathogenic TB diagnoses (*n* = 32) Clinical TB diagnoses (*n* = 16) Diagnosis basis: culture, AFS, Xpert, clinical criteria	• mNGS identified 67.23% infection cases within 3 days, while the conventional methods identified 49.58% infection cases for over 90 days. • For MTB BALF mNGS: Sen: 47.92% (23/48, 33.5–62.6%), similar to that of Xpert (45.83%, 22/48) and culture (46.81%, 22/47), but much higher than that of AFS (29.17%, 14/48); Spe: 98.39% (61/62, 90.2–99.9%).
Sun et al. (71)	• Type: B • Sample: smear-negative extrapulmonary samples (*n* = 208) • Pre-ATT samples (*n* = 129) • TB mNGS positive (*n* = 101) • Total TB diagnoses (*n* = 180) Clinical TB diagnoses (*n* = 180) Diagnosis basis: clinical criteria	• mNGS is superior for TB on smear-negative extrapulmonary specimens and could identify all possible pathogens within 48 h; mNGS positive rate was highest for TBM (84.44%, 38/45). • For MTB smear-negative extrapulmonary samples mNGS: Sen: 56.11% (101/180, 48.53–63.43%), Spe: 100% (28/28, 84.98–100.00%); PPV: 100% (101/101, 95.43–100.00%); NPV: 26.17% (28/107, 18.36–35.71%).
Liu et al. (72)	• Type: A • Sample: BALF (*n* = 322) • Pre-ATT TB samples (*n* = 142) Post-ATT TB samples (*n* = 69) • MTBC mNGS positive (*n* = 118) • Total TB diagnosis (*n* = 211) Diagnosis basis: culture, AFS, Xpert, clinical criteria	• Positive MTBC detection by mNGS was affected by Vitamin D, TB initial treatment/retreatment, erythrocyte sedimentation rate and cavity in chest imaging, but not by prior ATT within 3 months. • For MTBC pre-ATT BALF mNGS: Sen: 59.9% (85/142); while for MTBC post-ATT BALF mNGS: Sen 47.8% (33/69).
Lin et al. (103)	• Type: A + B • Sample: CSF (*n* = 50) • Pre-ATT samples (not mentioned) • MTBC mNGS positive (*n* = 20) • Total TB diagnosis (*n* = 34) Pathogenic TB diagnoses (*n* = 22) Clinical TB diagnoses (*n* = 12) Diagnosis basis: culture, AFS, Xpert, clinical criteria	• mNGS could rapidly detect MTBC in CSF, which could be used as an early diagnosis index of TBM. mNGS combined with MTB culture could increase the detection rate. • For MTB CSF mNGS: Sen: 58.8% (20/34), Spe: 100.0% (16/16).
Zhu et al. (104)	• Type: B • Sample: BALF (*n* = 78) • Lung tissue (*n* = 29) • Pre-ATT samples (not mentioned) • TB mNGS positive (*n* = 43) • Total TB diagnosis (*n* = 46)	• mNGS offers improved detection of MTB in BALF or lung tissue biopsy samples in sputum-scarce or smear-negative cases. • For MTB BALF mNGS: Sen: 90.63% (29/32, 73.83–97.55%), Spe: 97.83% (45/46, 87.03–99.89%), PPV: 96.67% (29/30, 80.95–99.83%); NPV: 93.75% (45/48, 81.80–98.37%); • For MTB lung tissue mNGS: Sen: 85.71% (12/14, 56.15–97.49%); Spe: 93.33% (14/15, 66.03–99.65%), PPV: 92.31% (12/13, 62.09–99.60%), NPV: 87.50% (14/16, 60.41–97.80%).

*Given the different focuses in different researches, the inclusive criteria varied. We classified the published mNGS literature into three types according to research focus and inclusive criteria: Type A (specific sample type was included, such as lung tissue, BALF, or CSF), Type B (specific pathogen was included, such as MTB), and Type C (comprehensive studies that enrolled all samples or patients in the research organizations). This review only includes the parts related to MTB infection.

*^$^It clearly defined the control groups: positive controls (bacterial/cryptococcal meningitis or viral meningoencephalitis) and negative control (auto-immune encephalitis).*

*Although there was no specific definition in other studies, similar study designs were carried. Therefore, control was not mentioned in this review.*

Pulmonary TB (PTB): Pulmonary involvement is common in MTB infection, affecting more than 75% of the total number of cases (1). Through the respiratory tract, multiple sample types can be taken, including bronchoalveolar lavage fluid (BALF), sputum, and lung tissue (45). Supported by the results of published studies, mNGS performed well in PTB: overall sensitivity was 44–59.9%, and specificity was 88.9–100%. Its sensitivity was superior to culture and AFS, while similar to Xpert. Lung tissue mNGS shows the peak sensitivity of 89% [95% confidence interval (CI), 51–99%] (30, 46), while sputum mNGS results are not superior to sputum culture (mNGS: 52%, 95% CI, 31–73%; culture: 61%, 95% CI, 39–80%), which may imply that mNGS shows better performance in sputum-negative PTB (30). BALF mNGS is more sensitive than blood mNGS in detecting bacteria (47), which may be due to the low number of detectable microbial sequences in the blood (41). While existing studies were partly contradictory against each other, inconsistent parts need to be viewed dialectically.

Extrapulmonary TB: MTB can be spread to invade extrapulmonary organs, including brain (48), bone, and joint (49). Therefore, corresponding samples [cerebrospinal fluid (CSF) (50, 51), joint fluid, and abscess (52)] can be used to ascertain the MTB infection in the specific organ. Studies on mNGS for tuberculous meningitis (TBM) have approved that mNGS showed excellent performance. The combination of CSF mNGS and routine methods increased the detection rate to 95.65% (51). Few systematic studies have been carried out on osteoarticular tuberculosis (OAT), but accurate detection results and good clinical outcomes have been reported in some cases (53, 54).

Disseminated TB: Although disseminated TB tends to endanger patients with immunodeficiency (55, 56), it is worth noting that immunocompetent patients may also suffer from that: a 51-year-old man had been repeatedly seeking for medical advice for hepatic mass without any discomfort for 2 years. With the help of mNGS of surgical specimens and BALF, he was eventually diagnosed with disseminated TB with systemic multi-organ involvement, including the lung, spine, mediastinum, liver, and prostate (57).

Additionally, the comparison of characteristic sequences can make the results accurate to the species level. mNGS complements the deficiency of traditional detections in identifying specific species in the MTB complex (MTBC), and it overcomes the difficulty in distinguishing between MTB and non-tuberculous *Mycobacteria* (NTM). Also, *Mycobacterium bovis* (58), *Mycobacterium abscessus* (59), and *Mycobacterium kansasii* (60) have been reported to be detected *via* mNGS.

### Co-infection and Comorbidity Detection

The mNGS overwhelms other pathogen detections in indicating co-infection. Given the strategy of casting a wide net, comprehensive detection and analysis can broaden the spectrum to bacteria, fungi, and viruses (51, 61, 62). Cell-free DNA sequencing was universally used in clinical trials and scientific studies for convenient transportation and stable performance (the data mentioned in this study were all generated from DNA sequencing). Although RNA sequencing was limited for being easily degradable and unstable (63), the combination of RNA sequencing is more complete and convincing for comprehensive diagnosis, which can get rid of missed diagnosis of RNA virus infection.

It is also promising that mNGS pipeline modification shows the potential to suggest underlying comorbidity. Predefining neural networks of chromosomal deletions or duplications, applying human reads generated from mNGS to map the reference human database, the Illumina platform assisted in diagnosing 36 cancer patients, of whom half had abnormal imaging findings (64). It takes full advantage of a large number of human host sequences and provides a new thread for mNGS application.

### Drug Resistance Detection

The emergence and prevalence of drug-resistant MTB strains is a major public health challenge. MDR-TB has recently grown at a rate of more than 20% a year (65). With the increasing proportion of MDR-TB strains against first-line TB drugs (RIF, INH, ethambutol, and pyrazinamide), there is a growing need for second-line drugs (fluoroquinolones and aminoglycosides) (66). It is calling for a test that is able to detect more mutations, especially for filling the vacancy of second-line drugs.

After targeted selection and preparation of antimicrobial resistance (AMR) genes, multiple drug resistance information can be reported through one detection (67). Currently, researchers are exploring and optimizing multiple platforms to support the rapid clinical decision-making toward MTB infection, and good results have been obtained on MiSeq, iSeq100 (67), and MinION (43, 44).

## Practical Considerations and Corresponding Solutions

### Improve Positive Rate

The MTB is an intracellular mycobacterial pathogen (68), and the cell wall is composed of high lipid content, especially a large amount of mycolic acid surrounding the outside of the peptidoglycan layer. They are the barriers blocking nucleic acid release (32). In addition, MTB has a long growth circle and paucibacillary pathogenicity (69), and there is a low bacterial load in non-abscess samples.

According to clinical symptoms and preliminary judgment, selecting appropriate samples is conducive to satisfactory results. Results generated from sterile body fluids (CSF, blood, and joint fluid) are more reliable for simple composition (32), while other samples (BALF and sputum) tend to have a more complicated composition of microorganisms but higher sensitivity and positive rate (30, 70). Also, the antituberculosis treatment (ATT) before sampling has an impact on the positive rate of MTB, and timely sampling is vital (50, 71, 72).

Optimization of the testing process helps to improve the positive rate. Enhanced preprocessing methods such as boiling and bead bashing may benefit nucleic acid extraction (73, 74). Enrichment strategies such as Finding Low Abundance Sequences by Hybridization (FLASH) (75) increase the sequences significantly. As for analyzing and interpreting, many researchers advised and used genus-specific read numbers ≥ 1 as the reporting threshold for MTB (76, 77). In addition, targeted NGS is a complementary method, and it enriches specific nucleic acids to get a greater depth of reads from a complex sample (78).

### Avoid Contamination

Amplification can amplify a low amount of contaminants over and over to detectable sequences (79), resulting in promiscuous reports. When sampling from the open airway or invasive operation, it is difficult to avoid contamination from respiratory preexist bacteria [*Streptococcus pneumoniae*, *Haemophilus influenzae*, *Moraxella catarrhalis* (80), and *Staphylococcus aureus* (81)], oral symbiotic microorganisms [*Streptococcus* (82) and *Candida albicans* (83)], and skin colonization colonies [*Cutibacterium acnes* (84) and *Moraxella osloensis* (85)]. Reagent and laboratory contamination are also notable. Excessive amplification of contaminated microorganisms will lead to false-positive results of contaminated microorganisms and also reduce the detection sensitivity of the pathogens. Therefore, the prejob training of sampling staff is needed to cut down the risk of sampling contamination. Blank controls and positive controls should be conducted to reveal the possible contaminating microorganisms (86).

Although the occurrence of MTB is relatively specific (53), it should be aware of false-positive results caused by contamination or cross-contamination. One MTB-positive mNGS report was later rejected by negative targeted PCR in residual samples, which was considered contamination during the mNGS procedures (61). It is also necessary to pay attention to the cross-contamination of samples from the same batch: if multiple samples in one run show low sequences of MTB, with one panel obtaining high sequences, the possibility of getting false-positive results should be alarmed. In one research, MTBC sequences were detected in 12/695 (1.5%) non-TB cases, while it is hard to interpret whether there is strong positive pollution in the same batch (30). Under this circumstance, it is of great importance to strictly implement quality control and promptly conduct clinical communication to decide whether to resample, re-sequence, or reanalyze.

### Interpret the Report

Being comprehensive also means it is hard to figure out the dominant pathogenic microbe. There is usually more than one suspected pathogen mentioned in one mNGS report. Therefore, how to determine the actual pathogenic microorganisms will be a big problem. First, the sequences are enriched *in vitro*, complex interactions of MTB and tissues enable the results to quantify the *in vivo* pathogenicity (87). Second, due to differences in extraction methods and gene stability, the sequence numbers of bacteria, fungi, and viruses in one report are not comparable. Third, common background microorganisms may also be the critical co-infectious opportunistic pathogens. In addition, negative results generated from low bacteria abundance samples are also worth noting, such as samples taken after ATT.

Therefore, raw data need to be carefully analyzed and evaluated under the codetermination of experimenters, analysts, clinicians, and, if necessary, the involvement of epidemiologists (88). In the case of a 77-year-old male patient with OAT, multiple suspect microorganisms were detected (53). After excluding laboratory contamination and virus with less clinical significance [*Torque teno virus* (TTV)] (89), the final diagnosis was confirmed as MTB infection.

### Optimize Cost Performance

Although mNGS has high sensitivity and specificity, compared with the mean cost of smear microscopy (US$13.31) and Xpert (US$17.37) (90), it has no advantage in cost and price. Given both test results and practical considerations [especially medical costs (91)], mNGS seems to be overqualified under ordinary circumstances. Sequencing results depend on the concentration of the sequences in the sample, so the cost and analysis time increase with increasing sequencing depth. After eliminating human host sequences, which make up almost 99% of the total sequence amount, less than 1% of the remaining sequences can be used for mNGS. Especially for low bacteria load in chronic extrapulmonary TB, the required cost and time for sequencing and analyzing were far beyond what many hospitals could bear (53).

On most occasions, mNGS is just a supplement to the routine tests and a vital research tool but not a conventional option. When the patient was exposed to MTB definitely and had typical symptoms, routine detections are preferred. mNGS may show better cost performance in the following circumstances: (1) the patient has unexplained manifestations beyond traditional assays or untypical symptoms such as fever, dyspnea, and elevated inflammatory markers (7); (2) the patient is strongly suspected of multi-pathogen infection; (3) the patient is in a critical condition, and the timely and comprehensive detection results are greatly needed.

### Expand Usage in Drug Resistance Detection

Although mNGS generates a considerable amount of data, it is limited in AMR gene detection in the current stage.

First, limitation owes to an inherent flaw of mNGS: short reads offered by Illumina are substantially not longer than 300 bp, tending to be shorter than the length of most mobile genetic elements; while Nanopore sequencers, which are the long-read sequencing approach, offer higher single-read error rates (92). Although researchers have proposed and verified that data integration and assembly can help to obtain drug resistance results (76, 93), it has not been widely used in clinical laboratories for complex data processing. Second, although mNGS shows potential in matching with multiple known drug resistance sites, new sites of drug resistance against second-line antituberculosis drugs still depend on further studies. To refine and expand the mutation catalog, scientists are carrying on studies of the relationship between phenotypic expression and genetic markers (2). Simultaneously, there are only a few specific sequences that can be detected, with fewer than 5 reads in half of the TB cases (30). It is also due to the low readings that drug resistance tests are not available on most occasions (94). Therefore, the detection of low abundance is a central challenge in clinical diagnostics (75). Enrichment strategies and target NGS are expected to improve the detection of AMR genes in addition to MTB detection. FLASH-NGS has enriched targeted sequences by > 100,000-fold and benefited detecting AMR genes of *S. aureus* and *Plasmodium falciparum* (75).

## Conclusion

The appearance of mNGS broadens our horizons, changing the hypothesis and validation process from “one-to-one” to “dozens-to-dozens”. Diagnostic mNGS is inclined to play an increasingly important role in the next decade (95). It provides a new method to distinguish between MTB and NTM, suggesting underlying co-infection or neoplastic disease. It also shows the potential of detecting AMR genes to guide clinical treatment. While considering the cost, it is deprecated to use for patients with mild or typical manifestations, for whom traditional targeted detection methods (Xpert) are more economical.

As the heat of the new technology is wearing off, practical problems are emerging: Will better detection reward a better outcome? Retrospective comparative studies have confirmed its effectiveness in diagnosis, but prospective clinical trials are lacking in knowing practical effects in the real world. We are looking forward to more high-quality studies to improve cost performance and standardize clinical application.

## Data Availability Statement

The original contributions presented in the study are included in the article/supplementary material, further inquiries can be directed to the corresponding author/s.

## Author Contributions

YaL, MJ, and YiL collected the literature and drafted the manuscript. AL and ZR provided guidance, revised the manuscript, and made equal contributions. All authors read and approved the final manuscript.

## Conflict of Interest

The authors declare that the research was conducted in the absence of any commercial or financial relationships that could be construed as a potential conflict of interest.

## Publisher’s Note

All claims expressed in this article are solely those of the authors and do not necessarily represent those of their affiliated organizations, or those of the publisher, the editors and the reviewers. Any product that may be evaluated in this article, or claim that may be made by its manufacturer, is not guaranteed or endorsed by the publisher.

## Abbreviations

AFS: acid-fast staining
ATT: antituberculosis treatment
BALF: bronchoalveolar lavage fluid
CI: Confident Intervals, all results mentioned in this chart were 95% CI
CSF: cerebrospinal fluid
PPV: positive predictive value
MTB: Mycobacterium tuberculosis
MTBC: MTB complex, including Mycobacterium tuberculosis, Mycobacterium canettii, Mycobacterium africanum, and Mycobacterium bovis, whose genome sequences showed high genomic similarity
NPV: negative predictive value
PCR: polymerase chain reaction
PTB: pulmonary tuberculosis
Sen: Sensitivity
Spe: Specifcity
TB: tuberculosis
TBM: tuberculous meningitis.
